# Imaging based risk factors for heart failure death in childhood dilated cardiomyopathy: a systematic review and meta-analysis

**DOI:** 10.3389/fcvm.2025.1568494

**Published:** 2025-04-09

**Authors:** Christina Street-de Palma, Zhia Lim, Ella Field, Juan Pablo Kaski, Gabrielle Norrish

**Affiliations:** ^1^ Centre for Paediatric Inherited & Rare Cardiovascular Disease, Institute of Cardiovascular Science, London, United Kingdom; ^2^ Centre for Inherited Cardiovascular Diseases, Great Ormond Street Hospital, London, United Kingdom

**Keywords:** dilated cardiomyopathy, childhood, outcomes, imaging, paediatric

## Abstract

**Background and aim:**

Dilated cardiomyopathy (DCM) is the most common heart muscle disease presenting in childhood and is associated with an increased risk of heart failure related death. In adult cohorts, imaging characteristics such as increased left ventricular dimensions or the presence of fibrosis on cardiac magnetic resonance imaging (MRI) have been shown to be associated with adverse outcomes. In contrast, the prognostic relevance of imaging characteristics in childhood cohorts remains unclear and predicting disease progression is challenging. The aim of this study was to perform a systematic literature review and meta-analysis of imaging characteristics associated with adverse outcomes in childhood DCM.

**Methods:**

PubMed, Embase, and Scopus databases were searched for original articles published in English from 1946 to July 2024 that included patients under 21 years with a confirmed diagnosis of DCM and primary or secondary end-points of heart failure death or equivalent event (heart transplantation or ventricular assist device implantation). Studies were excluded if imaging characteristics were not described.

**Results:**

Thirteen studies (1,348 patients) met the inclusion criteria. All but one study was retrospective and only one had a patient cohort of more than 100 patients. We identified four imaging risk factors that were evaluated in at least four studies and significantly associated with a heart failure end point in at least two; left ventricular end-diastolic diameter *Z* score (LVEDD) [pooled hazard ratio [HR] 1.43, 95% confidence interval [CI] 1.13–1.81, *p* = 0.003], left ventricular ejection fraction (LVEF) (pooled HR 0.8, 95% CI: 0.65–0.99, *p* = 0.04), LV fractional shortening (LVFS) and severe mitral regurgitation (MR) [pooled odds ratio (OR) 5.12, 95% CI: 1.18–22.19, *p* = 0.004]. Two small studies investigated the role of fibrosis on CMRI and did not report an association with adverse outcomes.

**Conclusions:**

A systematic review and meta-analysis of imaging risk factors predicting heart failure adverse events in childhood DCM was performed identifying three “major” risk factors; higher LVEDD, lower LVEF or LVFS and severe MR. The findings highlight a significant need for well-designed multicentre studies to investigate the role of imaging characteristics in predicting outcome in the paediatric population.

## Background

Dilated cardiomyopathy (DCM) is characterised by left ventricular dilation and systolic dysfunction and is the most common heart muscle disease presenting in childhood with an estimated incidence of 0.58–0.76 per 100,000 (1, 2). Population studies describing highly heterogeneous childhood populations have reported that early outcomes are poor with up to one third of patients dying or undergoing cardiac transplantation within 1 year of diagnosis (3, 4). Sudden death occurs less frequently than in adults but still with an estimated incidence of 1%–2% per year (5, 6). In adult cohorts, imaging characteristics such as left ventricular dimensions or the presence of fibrosis on cardiac magnetic resonance imaging (MRI) have been shown to be associated with an increased risk of heart failure or arrhythmic events (7, 8). However, it is currently unclear if similar or alternative imaging characteristics can predict outcomes in childhood disease. An ability to identify those individuals at high risk of poor outcomes, could lead to improved monitoring strategies and personalised treatment strategies.

The aim of this study was to perform a systematic literature review and meta-analysis of imaging characteristics associated with heart failure-related adverse outcomes in childhood DCM.

## Methods

### Study selection

The online databases Embase/Medline, PubMed/Medline were searched using the Medical Subject Headings (MeSH) terms “[(dilated cardiomyopathy OR DCM) AND (death OR cardiac transplant OR heart transplant OR outcomes OR heart failure OR predict) AND (paediatric OR pediatric OR child OR adolescent)]”. All searches were limited to original articles written in English and patients aged <21 years. This initial search strategy was supplemented by a manual search of the reference lists for included papers.

### Inclusion criteria

Studies reporting on a cohort of paediatric DCM patients with a primary or secondary end-point of heart failure death or equivalent event (heart transplantation or ventricular assist device implantation) were included. Studies with an end-point of arrhythmic events alone were excluded. Studies with no estimates of association between imaging risk factors and survival were excluded including case reports and letters. We excluded studies with a mixed cohort of adults and children with <50% of the population under 21 years of age without separate analysis of paediatric data. We also excluded studies reporting mixed cardiomyopathy phenotypes, that did not report outcomes specifically for patients with a DCM phenotype. As the study focused on imaging risk factors, studies exploring non-imaging risk factors, such as age at diagnosis or genetic influence, were excluded.

### Data collection

The title and abstract of all studies identified by the search strategy were reviewed by two independent researchers (CS and GN) to determine eligibility. All eligible studies were read in full by the same researchers. The following data were extracted from included studies: study design, country of study origin, study population size, patient demographics (age and sex), imaging risk factor, length of follow-up, univariate and/or multivariate Cox regression analysis, and event count data. A quality assessment for each included study was performed in line with the Joanna Briggs Institute (JBI) Critical Appraisal Checklist for Cohort Studies.

### Statistics

A random-effects meta-analysis was performed to combine the data from included studies reporting Cox regression analysis. The results from each Cox proportional hazards regression analysis were combined using the generic inverse-variance (IV) method. Survival analyses were not reported in all studies, and for these, count data were extracted to compute odds ratios considering the outcome as a dichotomous variable. Pooled ORs were calculated using the Mantel-Haenszel method. We report the pooled HRs or ORs for each risk factor available with a 95% CI for each summary estimate. The percentage of variability in estimates due to heterogeneity between the studies was reported as the I² score. A significance level of 5% (*p* = <0.05) was used for analysis. The analysis was performed on Review Manager (version 5.3).

## Results

### Study selection

**Figure 1:**
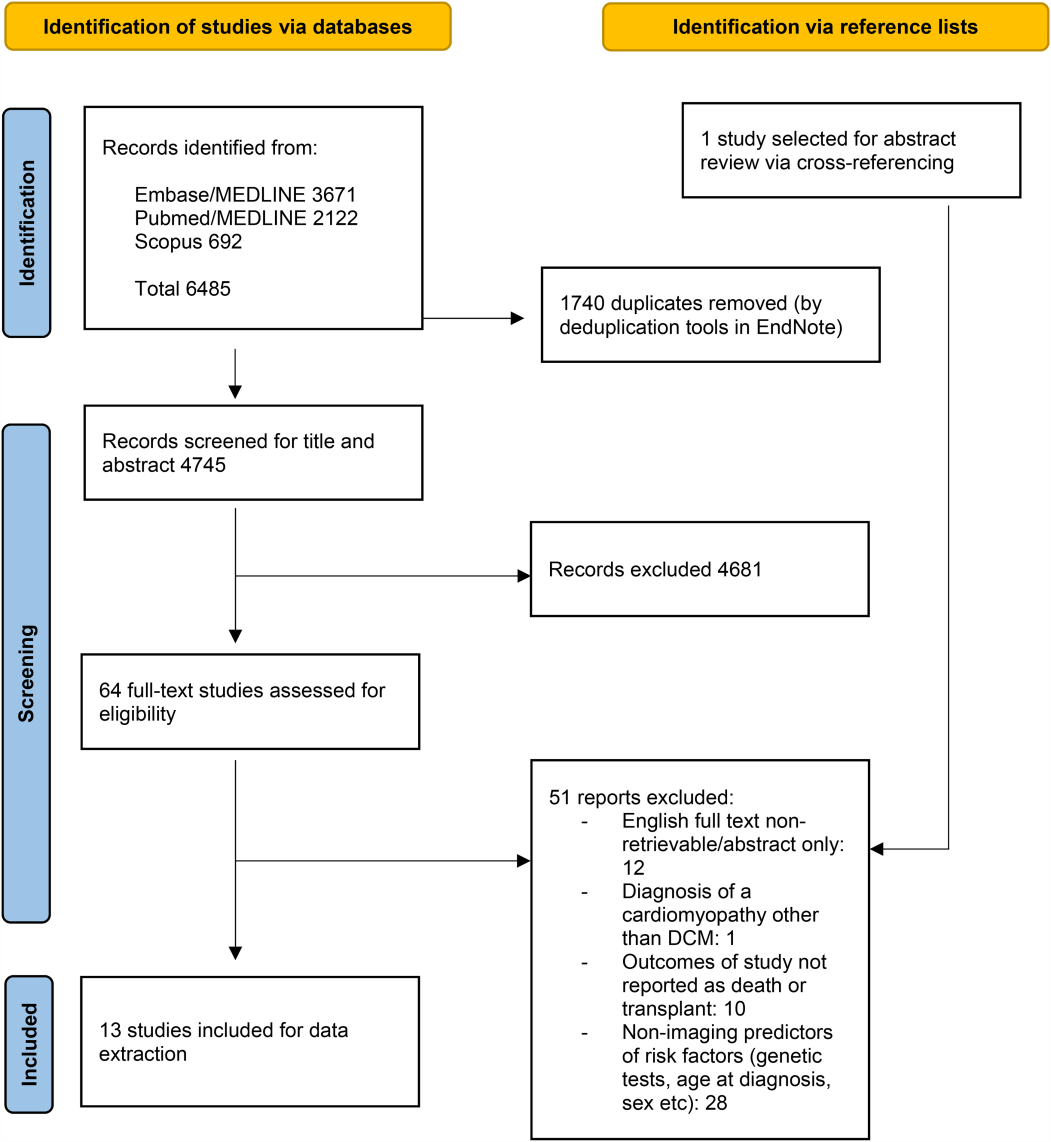
Identification of the studies included for data synthesis.

Figure 1 summarises the search result. Briefly, the preliminary search identified 4,745 unique studies. 4,681 of these studies were excluded after reading the titles and abstracts. The full text was read for the remaining 64 articles to assess for eligibility. This excluded a further 51 articles. The reasons for exclusion are described in Figure 1. Data was extracted from the 13 studies that met inclusion criteria (Table 1).

**Table 1:** All included articles (*n* = 13).

				Age (years)			Follow up (months)		
References	Type	Country	N of patients	Mean	Median (IQR)	Male (%)	Mean	Median	Imaging modality
Al-Wakeel-Marquard et al. (9)	Cohort retrospective	Netherlands	17	ND	6.7 (0.2−13.0)	53	ND	32 (12–46)	MRI
Muscogiuri et al. (10)	Cohort retrospective	Italy	15	8 ± 6	ND	40	ND	17	MRI
Capone et al. (11)	Cohort retrospective	USA	48	9 ± 7	ND	62	ND	24	Echo
Fernandes et al. (12)	Cohort retrospective	Canada	42	7.4 ± 6.9	ND	65	ND	9 (1–200)	Echo
Ishii et al. (13)	Cohort retrospective	Canada	57	4.5 ± 5.8	ND	51	ND	ND	Echo
Kantor et al. (14)	Registry	USA	794	ND	1–2	53	ND	8.6 (6.0–10.4)	Echo
Lewis et al.(15)	Cohort retrospective	USA	72	3.7 ± 0.7	ND	ND	3.6 ± 0.5	ND	Echo
Limongelli et al. (16)	Cohort retrospective	Italy	48	ND	17 (13–19)	35	4.4 ± 3.6	ND	Echo
McMahon et al. (17)	Cohort retrospective	USA	54	ND	3 (1.3–13.7)	46	ND	21 (6–42)	Echo
Mondal et al. (18)	Cohort retrospective	Canada	48	7.7 ± 6.6	ND	48	ND	ND	Echo
Patange et al. (19)	Cohort retrospective	USA	49	ND	1.25 (0.1–17)	43	ND	12 (2d-18yrs)	Echo
Raj et al. (20)	Cohort retrospective	India	57	ND	13 (7–16)	58	ND	ND	MRI/Echo
Garcia-Canadilla et al. (21)	Cohort retrospective	Spain	47	4.09 ± 5.5	ND	53	ND	ND	Echo

### Study characteristics

The 13 included studies are described in Table 1. All but one study was conducted in Western Europe or North America. The majority (*n* = 12) were retrospective studies with small patient cohorts of less than 75 patients. One study reported data from a large prospective registry (*n* = 794 patients) (14). The diagnostic criteria for DCM varied across studies (Supplementary Table S1). Of the included studies, 11 (85%) reported echocardiography-based risk factors and 2 (15%) cardiac MRI risk factors for heart failure adverse events. All included studies investigated an association between baseline imaging characteristics and outcomes whilst two studies investigated an association between serial imaging risk factors and adverse events (13, 14). In total, 82 individual imaging risk factors were described, but only 23 were investigated in more than one study (Supplementary Table S2).

### Imaging based risk factors

Due to the large numbers of risk factors only looked at in single studies we divided the reported risk factors into two main groups for the purpose of analysis: probable risk factors (defined as being investigated as a potential risk factor in at least four studies and significantly associated with the primary outcomes in at least two univariate or multivariate analyses); and possible risk factors (defined as being significantly associated with the primary outcomes in at least one univariate or multivariate analysis study).

#### Probable echocardiographic risk factors

##### Left ventricular end-diastolic diameter (LVEDD) *z*-score

**Figure 2:**
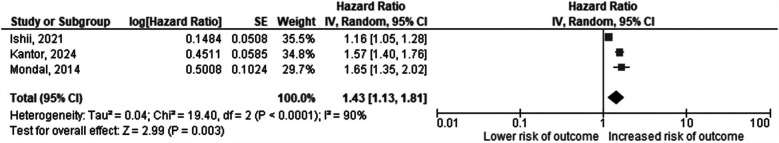
Hazard ratios for increasing LVEDD-z score as a predictor of death or cardiac transplantation. The squares represent effect estimates from each study while the diamond shows the pooled result. The bars represent the upper and lower 95% CIs. Hazard ratios with CIs > 1 indicate a significant association with death or cardiac transplantation. IV, inverse-variance method; CI, confidence interval.

Eight studies investigated LVEDD *z*-score on echocardiography at baseline as a potential risk factor for heart failure event in children with DCM (11, 13–15, 17–19, 21). A significant association with the primary outcome was shown in 4 studies, 3 of which reported survival analyses (13, 14, 18). The pooled HR was 1.43 (95% CI: 1.13–1.81, *p* = 0.003, *I*^2^ = 90%) (Figure 2). Two studies investigated the predictive value of serial LVEDD Z score measurements and reported an association between progressive increase in LVEDD Z score and heart failure adverse outcomes (13, 21).

##### Left ventricular systolic function

**Figure 3:**
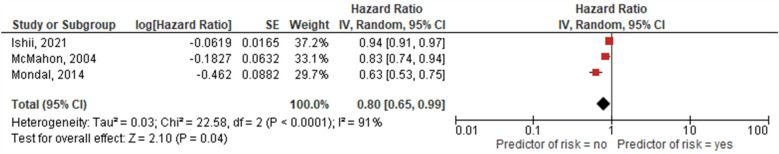
Hazard ratios for higher LVEF as predictor of death or cardiac transplantation. The squares represent effect estimates from each study while the diamond shows the pooled result. The bars represent the upper and lower 95% CIs. Hazard ratios with CIs > 1 indicate a significant association with death or cardiac transplantation. IV, inverse-variance method; CI, confidence interval.

Six studies assessed the prognostic value of reduced LV ejection fraction (EF) on echocardiography at baseline for heart failure events all of which reported a significant association (11, 13, 16–18, 20). Three studies (13, 17, 18) performed survival analysis with a pooled HR of 0.8 (95% CI: 0.65–0.99, *p* = 0.04, *I*^2^ = 91%) (Figure 3). Five studies investigated LV fractional shortening (FS) as assessed using echocardiography M mode as a risk factor for heart failure event (11, 13, 15, 16, 19). Two of these studies reported a significant relationship between lower LV FS and risk of a heart failure event (11, 16) neither of which reported survival analysis data. One study investigated the predictive role of changes in LVFS over time and reported an increase in LVFS to be inversely associated with the risk of heart failure event (14).

##### Mitral regurgitation

**Figure 4:**
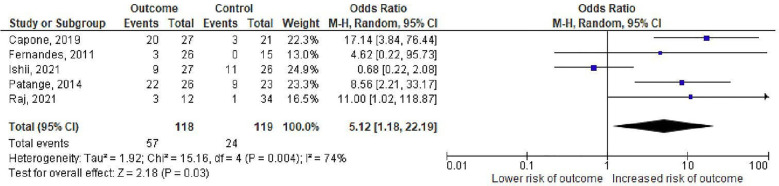
Odds ratios for mitral regurgitation as a predictor of death or cardiac transplantation. The squares represent effect estimates from each study while the diamond shows the pooled result. The bars represent the upper and lower 95% CIs. Odds ratios with CIs > 1 indicate a significant association with death or cardiac transplantation. M–H: Mentel-Haenzsel method; CI: confidence interval.

The degree of mitral regurgitation on echocardiography was considered as a risk factor in 5 studies of which 3 found a significant relationship between moderate/severe mitral regurgitationand the outcome (11–13, 18, 19). The pooled OR estimate for moderate/severe mitral regurgitation was 5.12 (95% CI: 1.18–22.19, *p* = 0.004, I2 = 74%) (Figure 4).

#### Possible echocardiographic risk factors

A further 17 echocardiographic imaging risk factors were described, of these 8 were investigated in a single study (Table 2).

**Table 2:** A summary of possible echocardiographic risk factors for adverse outcomes in paediatric DCM.

Category	Risk factor	Number of Studies	Findings	Significance (*p*-value)
Left ventricular dimensions (11, 13–16, 19, 20)	LVEDD	4 (10, 15, 16, 20)	Only one study reported a statistically significant association with the primary outcome, (*p* = <0.001)	Significant (*p* = <0.001)1 (6)
	LVESD z-score	3 (11, 13, 19)	Only one study reported a significant association (*p* = <0.002) (11)	Significant (*p* = <0.002) (11)
	IVS thickness	1 (11)	significantly associated with heart failure adverse events (*p* = <0.001) (11)	Significant (*p* = <0.001) (11)
	LVPWT:LVEDD z-score	1 (14)	significantly associated with heart failure events (*p* = 0.02) (14)	Significant (*p* = 0.02) (14)
Left ventricular geometrics (11, 13)	Sphericity index	2 (11, 13)	One study reported a significant association with heart failure adverse events (*p* = 0.005) (11)	Significant (*p* = 0.005) (11)
	Sphericity index z-score	2 (11, 13)	One study found a significant relationship after adjustment for age, sex, and body size (*p* = 0.01) (11)	Significant (*p* = 0.01) (11)
Markers of diastolic dysfunction (11, 13, 15, 17)	TDI LV eʹ velocity	1 (11)	Reduced LV eʹ velocity was significantly associated with death or transplant.	Significant (*p* = 0.001) (11)
	E-wave deceleration time	1 (11)	Short E-wave deceleration time was significantly associated with poor outcomes.	Significant (*p* = 0.02) (11)
	Mitral septal Ea	1 (16)	Lower mitral septal Ea was significantly associated with poor outcomes.	Significant (*p* = 0.001) (16)
	Tricuspid Ea velocity	1 (17)	Lower tricuspid Ea velocity was associated with death or cardiac transplant.	Significant (*p* = 0.03) (17)
Ventricular arterial coupling (11)	Arterial elastance, LV elastance, VA coupling ratio	1 (11)	All three parameters were significantly associated with the primary outcome.	Significant (*p* = 0.002, 0.001, 0.001) (11)
Right heart measurements (16, 17)	RVFAC	2 (17)	RVFAC was significantly associated with adverse outcomes.	Significant (*p* = 0.02) (17)
	Estimated systolic pulmonary pressure	1 (16)	Increased systolic pulmonary pressure was significantly associated with outcomes.	Significant (*p* = 0.002) (15)

LVESD, left ventricular end-systolic dimension; IVS, interventricular septum; LVPWT, left ventricular posterior wall thickness; LVEDD, left ventricular end-diastolic dimension; TDI, tissue doppler imaging; LV eʹ: left ventricular early diastolic velocity; Ea, early diastolic mitral annular vVelocity; RVFAC, right ventricular fractional area change; VA Coupling, ventricular-arterial coupling.

##### Cardiac magnetic resonance imaging

Only two studies explored the role of cardiac MRI based imaging risk factors for heart failure events (9, 10). Al-Wakeel-Marquard et al. found no association between midwall fibrosis and the outcome (*p* = 0.637) (9). Muscogiuri et al. reported that the frequency of major adverse cardiac events was higher in patients with diffuse subendocardial LGE (38% vs. 14%), but this was not statistically significant (10).

## Discussion

This study is, to our knowledge, the first systematic review of potential imaging risk factors for heart failure adverse events for childhood onset DCM. We have identified 4 probable risk factors at baseline associated with an increased risk of heart failure related adverse outcome: LVEDD Z score, LVEF, LVFS and the presence of moderate/severe mitral regurgitation. Although a large number of potential imaging-based risk factors were identified, the lack of consistent definitions and well-designed, large- population studies means that the evidence base for individual risk factors is limited. This highlights a significant unmet need for standardised imaging criteria and larger, well-structured studies to establish robust risk stratification models. Addressing these gaps is crucial for improving early risk prediction and guiding clinical management strategies for children with DCM.

### Predicting outcomes in childhood DCM

Systematic description of the imaging phenotype is essential for both the diagnosis and management of childhood onset DCM (22). However, it remains unclear if imaging characteristics can be used to identify groups of patients at the highest risk of adverse events (23). This systematic review has identified 4 echocardiography-based imaging parameters that are likely to be associated with heart failure events including higher left ventricular diastolic dimensions (LVEDD), measures of poorer LV systolic function (LVFS and LVEF) and moderate or severe mitral regurgitation. These imaging characteristics typically co-exist in individual patients, but it is beyond the scope of this study to determine the interaction and additive effect of different imaging risk factors on patient outcomes. A large number of other echocardiography-based risk factors were assessed in a single or small number of studies. It is likely that a number of these imaging parameters are important for outcome prediction in childhood DCM but there is insufficient evidence to support their use at this time. Large, well designed, multi-centre studies are needed to systematically evaluate the role of individual imaging parameters in childhood DCM.

The natural history of children presenting with DCM is recognised to be highly variable with up to a third experiencing “remodelling” and normalisation of LV dimensions and systolic function over follow up (24). Only two of the identified studies investigated the role of serial imaging in outcome prediction, both of which found that changes in LV dimensions or function over time may be more powerful predictors of long-term outcomes compared to baseline measurements (13, 14). One study also suggested that not only the degree of recovery but also the speed by which remodelling occurs may be important for predicting the natural history of disease (13). This is perhaps not surprising given that childhood is a time of significant somatic growth and remodelling; this is not accounted for when the phenotype is assessed at a single time point (24). This study focused on imaging-based risk factors for DCM but it is well-recognised that additional non-imaging-based characteristics are likely to be important including underlying aetiology, family history, symptoms, presence of malignant ventricular arrhythmias and blood biomarkers (e.g., NT-proBNP) (5, 6, 25). Future, larger cohort studies are needed to describe the changing phenotype over time in childhood onset DCM and explore the interaction between individual imaging and non-imaging-based risk factors.

### Role of cardiac MRI in childhood DCM

In adult DCM cohorts, cardiac MRI is widely used for diagnostic and prognostic purposes. Patterns of late gadolinium enhancement (LGE) have been shown to be associated with specific aetiologies such as sub-epicardial distribution in post-myocarditis, septal mid-wall in LMNA and ring-like in DSP and FLNC carriers (26). The extent and distribution of LGE have also been shown to be a risk factor for both heart failure and arrhythmic events (7, 8). Only two studies were identified that explored the role between LGE and outcomes in childhood onset disease both of which had small patient cohorts (less than 20 patients) (9, 10). Although the frequency of events was higher in those with LGE in one of the studies, a significant association was not seen. The current scarcity of evidence prevents robust conclusions about the role of LGE in childhood DCM being made. The lack of studies may in some part be explained by challenges in scanning younger patients, including difficulties in obtaining high-quality images due to a higher heart rate or need for sedation meaning cardiac MRI may be used less routinely than in adult practice. Such difficulties could be overcome by large multi-centre studies, which are urgently required to systematically determine the role of cardiac MRI in for outcome prediction in childhood DCM.

### Limitations

The number of studies included in this systematic review and meta-analysis is small, and all but one was a retrospective cohort study. It is therefore limited by inherent problems of retrospective studies including missing and incomplete information. Although the most common cardiomyopathy presenting in childhood, DCM is a rare disease, and all but one of the included studies were small with less than 75 participants. Small studies are more prone to variability, which can lead to overestimation of effect sizes and reduce the precision of pooled estimates. This limitation may contribute to the observed high heterogeneity in some analyses, indicating that differences in sample size and study design may account for some of the variability in results. Pooled estimates for average HR's and OR's are reported; however the low number of events and small number of studies means the precision of estimates is low as reflected by the width of CI for pooled ratios. The reliability of the meta-analysis is therefore affected and these estimates should be interpreted with caution. We did not exclude studies based on sample size and, as most cohorts were small, we were not able to perform sensitivity analyses to investigate the effect of sample size on analysis findings. Multicentre collaborations and large patient cohorts should be prioritised to enhance the generalizability and reliability of findings.

A key limitation of this meta-analysis is the substantial heterogeneity observed across studies, with I² values reaching 90% in some meta-analyses. This high heterogeneity likely arises from multiple factors, including differences in study populations (e.g., age at diagnosis, aetiology, severity of disease),variable definitions of imaging parameters and operator dependent differences in image acquisition and interpretation Random-effects modelling was used to account for this variability but the wide confidence intervals in estimates suggest caution should be used in interpreting pooled effect sizes. Future research should focus on standardising definitions and methodologies to improve comparability across studies.

## Conclusion

A systematic review and meta-analysis of imaging risk factors to predict heart failure adverse events in childhood DCM has identified four “major” risk factors; higher LVEDD, lower LVEF or LVFS, and severe MR. A large number of other potential or “minor” risk factors have been described in single studies. The evidence base supporting the role of individual imaging characteristics is limited by the low number of studies describing small and heterogenous patient populations. Well-designed multicentre studies are required to investigate the role of imaging characteristics in predicting outcome for childhood DCM.

## Data Availability

The original contributions presented in the study are included in the article/Supplementary Material, further inquiries can be directed to the corresponding author.

## References

[1] Arola A Jokinen E Ruuskanen O Saraste M Pesonen E Kuusela A-L Epidemiology of idiopathic cardiomyopathies in children and adolescents. A nationwide study in Finland. Am J Epidemiol. (1997) 146(5):385–93. 10.1093/oxfordjournals.aje.a009291 9290498

[2] Lipshultz SE Sleeper LA Towbin JA Lowe AM Orav EJ Cox GF The incidence of pediatric cardiomyopathy in two regions of the United States. N Engl J Med. (2003) 348(17):1647–55. 10.1056/NEJMoa021715 12711739

[3] Andrews RE Fenton MJ Ridout DA Burch M. New-onset heart failure due to heart muscle disease in childhood: a prospective study in the United Kingdom and Ireland. Circulation. (2008) 117(1):79–84. 10.1161/circulationaha.106.671735 18086928

[4] Alexander PM Daubeney PE Nugent AW Lee KJ Turner C Colan SD Long-term outcomes of dilated cardiomyopathy diagnosed during childhood: results from a national population-based study of childhood cardiomyopathy. Circulation. (2013) 128(18):2039–46. 10.1161/circulationaha.113.002767 24036608

[5] Pahl E Sleeper LA Canter CE Hsu DT Lu M Webber SA Incidence of and risk factors for sudden cardiac death in children with dilated cardiomyopathy: a report from the pediatric cardiomyopathy registry. J Am Coll Cardiol. (2012) 59(6):607–15. 10.1016/j.jacc.2011.10.878 22300696 PMC3280885

[6] Bharucha T Lee KJ Daubeney PE Nugent AW Turner C Sholler GF Sudden death in childhood cardiomyopathy: results from a long-term national population-based study. J Am Coll Cardiol. (2015) 65(21):2302–10. 10.1016/j.jacc.2015.03.552 26022819

[7] Di Marco A Anguera I Schmitt M Klem I Neilan TG White JA Late gadolinium enhancement and the risk for ventricular arrhythmias or sudden death in dilated cardiomyopathy: systematic review and meta-analysis. JACC Heart Fail. (2017) 5(1):28–38. 10.1016/j.jchf.2016.09.017. Erratum in: JACC Heart Fail. 2017 Apr;5(4):316. doi: 10.1016/j.jchf.2017.02.006 28017348

[8] Chen W Qian W Zhang X Li D Qian Z Xu H Ring-like late gadolinium enhancement for predicting ventricular tachyarrhythmias in non-ischaemic dilated cardiomyopathy. Eur Heart J Cardiovasc Imaging. (2021) 22(10):1130–8. 10.1093/ehjci/jeab117 34160025

[9] Al-Wakeel-Marquard N Seidel F Kühnisch J Kuehne T Berger F Messroghli DR Midwall fibrosis and cardiac mechanics: rigid body rotation is a novel marker of disease severity in pediatric primary dilated cardiomyopathy. Front Cardiovasc Med. (2022) 8:810005. 10.3389/fcvm.2021.810005 35252369 PMC8891497

[10] Muscogiuri G Ciliberti P Mastrodicasa D Chinali M Rinelli G Santangelo TP Results of late gadolinium enhancement in children af by dilated cardiomyopathy. Front Pediatr. (2017) 5. 10.3389/fped.2017.00013 28220144 PMC5292614

[11] Capone CA Lamour JM Lorenzo J Tria B Ye K Hsu DT Ventricular arterial coupling: a novel echocardiographic risk factor for disease progression in pediatric dilated cardiomyopathy. Pediatr Cardiol. (2019) 40:330–8. 10.1007/s00246-018-2021-6 30415380

[12] Fernandes FP Manlhiot C McCrindle BW Mertens L Kantor PF Friedberg MK. Usefulness of mitral regurgitation as a marker of increased risk for death or cardiac transplantation in idiopathic dilated cardiomyopathy in children. Am J Cardiol. (2011) 107(10):1517–1521. 10.1016/j.amjcard.2011.01.030 21377646

[13] Ishii R Steve Fan CP Mertens L Manlhiot C Friedberg MK. Longitudinal prediction of transplant-free survival by echocardiography in pediatric dilated cardiomyopathy. Can J Cardiol. (2021) 37(6):867–876. 10.1016/j.cjca.2020.12.010 33347978

[14] Kantor PF Shi L Colan SD Orav EJ Wilkinson JD Hamza TH Progressive left ventricular remodeling for predicting mortality in children with dilated cardiomyopathy: the pediatric cardiomyopathy registry. J Am Heart Assoc. (2024) 13(2). 10.1161/JAHA.121.022557 PMC10926795 38214257

[15] Lewis AB. Prognostic value of echocardiography in children with idiopathic dilated cardiomyopathy. Am Heart J. (1994) 128(1):133–136. 10.1016/0002-8703(94)90019-1 8017266

[16] Limongelli G Pacileo G Ancona R Eusebio G D'Andrea A Romano M Clinical course and risk profile in adolescents with idiopathic dilated cardiomyopathy. Am J Cardiol. (2010) 105(5):716–20. 10.1016/j.amjcard.2009.10.055 20185022

[17] McMahon CJ Nagueh SF Eapen RS Dreyer WJ Finkelshtyn I Cao X Echocardiographic predictors of adverse clinical events in children with dilated cardiomyopathy: a prospective clinical study. Heart. (2004) 90(8):908–15. 10.1136/hrt.2003.020966 15253966 PMC1768368

[18] Mondal T Slorach C Manlhiot C Hui W Kantor PF McCrindle BW Prognostic implications of the systolic to diastolic duration ratio in children with idiopathic or familial dilated cardiomyopathy. Circ Cardiovasc Imaging. (2014) 7(5):773–780. 10.1161/CIRCIMAGING.114.002120 25140066

[19] Patange A Thomas R Ross RD. Severity of mitral regurgitation predicts risk of death or cardiac transplantation in children with idiopathic dilated cardiomyopathy. Pediatr Cardiol. (2014) 35(2):232–238. 10.1007/s00246-013-0764-7 23917522

[20] Raj S Kothari R Arun Kumar N Sigamani A Raj V. T1 mapping and conditional survival in paediatric dilated cardiomyopathy with advanced heart failure. Cardiol Young. (2021) 31(12):1938–1942. 10.1017/S1047951121001268 33827738

[21] Garcia-Canadilla P Sanchez-Martinez S Martí-Castellote PM Slorach C Hui W Piella G Machine-learning–based exploration to identify remodeling patterns associated with death or heart-transplant in pediatric-dilated cardiomyopathy. J Heart Lung Transplant. (2022) 41(4):516–26. 10.1016/j.healun.2021.11.020 35063339

[22] Arbelo E Protonotarios A Gimeno JR Arbustini E Barriales-Villa R Basso C 2023 ESC guidelines for the management of cardiomyopathies: developed by the task force on the management of cardiomyopathies of the European Society of Cardiology (ESC). Eur Heart J. (2023) 44(37):3503–626. 10.1093/eurheartj/ehad194 37622657

[23] Moscatelli S Leo I Bianco F Borrelli N Beltrami M Garofalo M The role of multimodality imaging in pediatric cardiomyopathies. J Clin Med. (2023) 12(14):4866. 10.3390/jcm12144866 37510983 PMC10381492

[24] Everitt MD. When and how does dilated cardiomyopathy recover in children? Prog Pediatr Cardiol. (2021) 62:101400. 10.1016/j.ppedcard.2021.101400

[25] Hauser JA Demyanets S Rusai K Goritschan C Weber M Panesar D Diagnostic performance and reference values of novel biomarkers of paediatric heart failure. Heart. (2016) 102(20):1633–9. 10.1136/heartjnl-2016-309460 27220692

[26] Augusto JB Eiros R Nakou E Moura-Ferreira S Treibel TA Captur G Dilated cardiomyopathy and arrhythmogenic left ventricular cardiomyopathy: a comprehensive genotype-imaging phenotype study. Eur Heart J Cardiovasc Imaging. (2020) 21(3):326–36. 10.1093/ehjci/jez188. PMID: 31317183 31317183

