# Supplementary material for: Imaging based risk factors for heart failure death in childhood dilated cardiomyopathy: a systematic review and meta-analysis
Source: Front Cardiovasc Med. 2025 Apr 9;12:1568494. doi: 10.3389/fcvm.2025.1568494 (PMC12014537; doi:10.3389/fcvm.2025.1568494)
Supplement: Supplementary file 1 [file Datasheet1.pdf]

Supplementary table 1. Inclusion and exclusion criteria used for DCM patients in the selected studies.

**References**	**Exclusion**	**Inclusion**
Al-Wakeel-Marquard *et al.* 2022	Myocarditis, systemic disease with cardiac involvement, combination with other congenital heart diseases, contraindications to perform CMR.	Under 21 years of age with diagnosis of primary DCM.
Muscogiuri *et al.* 2017	DCM secondary to other cardiac conditions and with glomerular filtration rate <30ml/min/1.73m^2, or frequent ventricular arrhythmias.	LV end-diastolic dimension *Z* score >2 and LV EF <50%.
Capone *et al.* 2019	Congenital heart disease and patients on mechanical circulatory support or listed for transplant at the time of presentation.	21 years old and under. DCM LVEDD Z score>2 and LVEF <50%.
Fernandes *et al.* 2011	Receiving/received anthracyclines, congenital structural heart disease, undergone mitral surgery, or diagnosed with acute myocarditis, LV noncompaction, hypertrophic cardiomyopathy, or LV dysfunction after cardiac arrest.	DCM LVEDD Z score>2 and LVEF 50%.
Ishii *et al.* 2021	Anatomic heart disease, mitral valve surgery, or hypertrophic or LV noncompaction cardiomyopathy.	Under 18 years old. DCM LVEDD Z score>2 and LVEF 50%.
Kantor *et al.* 2024	Cardiomyopathies from systemic diseases or in association with malformation syndromes.	Under 18 years old. DCM LVEDD Z score>2 and LVFS Z score<2.0.
Lewis *et al.*1994	Metabolic, neuromuscular, or collagen-vascular disorders and patients with clinical or laboratory findings consistent with myocarditis.	Dilated, poorly contracting left ventricles in the absence of any congenital or acquired structural or functional cardiac abnormality.
Limongelli *et al.* 2010	Patients with clinical onset of LV systolic dysfunction at <13 years or >19years of age.	LVEF <45% with LVEDD>2 for an age-body surface area matched population.
McMahon *et al.* 2004	Poor echocardiographic windows, sub-optimal TD imaging, or incomplete acquisition of study data.	LVEF <40% with LVEDD>2 Z score.
Mondal *et al.* 2014	Patients with structural heart disease or those who had undergone previous surgery.	LVEDD>2 Z score and LVEF<50%.
Patange *et al.* 2014	DCM secondary to other causes, DCM secondary to viral myocarditis and the presence of a coexisting congenital or structural cardiac disease.	Under 18 years old. LVEDD>2 and FS of <28%.
Raj *et al.* 2021	Patients with heart failure and left ventricular dysfunction due to other cardiomyopathies or structural heart disease.	LVEDD>2 Z score and LVEF<45% with the absence of structural heart disease.
Garcia-Canadilla *et al.* 2022	Anatomic heart disease, pacing, mitral surgery, or LV noncompaction.	LVEDD>2 Z score and LVEF<50%.

Supplementary table 2: List of all imaging parameters investigated in included studies

**Risk factor**	**Number of studies**	**Reference**
Left Ventricular End-Diastolic Diameter (LVEDD)	4	11, 15, 16, 20
LVEDD Z Score	6	11, 13, 14, 15, 19, 21
Left Ventricular End-Systolic Diameter (LVESD)	1	11
LVESD Z Score	3	11, 13, 19
Left Ventricular Posterior Wall Diameter Z Score (LVPWD)	1	13
Left Ventricular Posterior Wall Thickness Z Score (LVPWT)	2	14, 19
LVPWT:LVEDD Z Score	1	14
Interventricular Septum (IVS)	1	11
IVS Z Score	1	11
Posterior Wall Thickness (PWT)	1	11
PWT Z Score	1	11
PWT:LVEDD	1	11
Sphericity Index	2	11, 13
Sphericity Index Z Score	2	11, 13
Left Atrial Size Indexed to Body Surface Area (LA BSA)	1	16
Left Ventricular (LV) Volume	1	19
LV Mass	1	15
LV Mass Z Score	1	15
Mitral S' Velocity Z Score	1	13
Mitral E' Velocity Z Score	1	13
Mitral E/e'	1	13
Mitral E/A	1	13
Mitral E Deceleration Time	1	13
Isovolumic Relaxation Time	1	13
Pulmonary Venous Systolic Wave	1	13
Pulmonary Venous Diastolic Wave	1	13

Pulmonary Vein Atrial Velocity	1	13
Pulmonary Artery Hypertension	1	20
Peak E Velocity	1	17
Peak A Velocity	1	17
Acceleration Time	1	17
Deceleration Time	1	17
Right Ventricular End-Diastolic Diameter Z Score (RVEDD)	1	13
Fractional Change Area	1	13
Tricuspid Annular Plane Systolic Excursion Z Score (TAPSE)	1	13
Tricuspid S’ Z Score	1	13
Tricuspid E’ Z score	1	13
Right Ventricular Myocardial Performance Index (RV MPI)	1	13
Tricuspid Regurgitation (TR)	1	13
TR Grade	1	13
Right Ventricular Tei Index (RV TEI)	1	17
Right Ventricular Fractional Area Change (RVFAC)	1	17
Tricuspid E/A Ratio	1	13
TV e’	1	13
Mitral Valve Regurgitation Grade (MV)	5	11, 12, 13, 19, 20
Restrictive Mitral Filling Pattern	1	16
Systolic Pulmonary Pressure	1	16
Tricuspid Annulus Peak Systolic Excursion	1	16
Mitral Septal EA	2	11, 17
EA Z Score	1	11
Septal E/Ea	1	16
Septal Ea	1	16
Septal Aa	2	16, 17

Mitral Septal Aa	1	11
Aa Z Score	1	11
Mitral Septal Sa	2	11, 17
Mitral Lateral Sa	1	17
Sa Z Score	1	11
Lateral Ea	2	16, 17
Lateral Aa	2	16, 17
Lateral E/Ea	1	16
Tricuspid Ea	2	16, 17
Tricuspid Aa	2	16, 17
Tricuspid E/Ea	1	16
Tricuspid Sa	1	17
Septal s’	1	13
Ejection Fraction (EF)	6	11, 13, 15, 16, 17, 20
EF Z Score	3	11, 13, 21
Shortening Fraction (SF)	3	11, 16, 19
SF Z Score	3	11, 13, 14
Global Longitudinal Strain (GLS)	1	21
Left Ventricular Myocardial Performance Index (LV TEI)	2	11, 17
Systolic to Diastolic Ratio (SD)	1	11
Arterial Elastance	1	9
LV Elastance	1	9
Ventricular-Arterial Coupling Ratio (VA)	1	9
Myocardial Fibrosis	1	10
